# Indonesian medical frontliners during the coronavirus disease 2019 pandemic: Have we been protecting them enough?

**Published:** 2021-07-30

**Authors:** Gilbert Lazarus, Markus Meyer, Markus Depfenhart, Angela Kimberly Tjahjadi, Santi Rahayu Dewayanti, Iwan Dakota, Bambang Budi Siswanto

**Affiliations:** ^1^Faculty of Medicine, Universitas Indonesia, Jakarta, Indonesia; ^2^North-West University, Potchefstroom, South Africa; ^3^Faculty of Medicine, Venlo University B.V, Venlo, Netherlands; ^4^Department of Pulmonology, National Cardiovascular Centre Harapan Kita, Faculty of Medicine Universitas Indonesia, Jakarta, Indonesia; ^5^Department of Cardiology and Vascular Medicine, Faculty of Medicine, Universitas Indonesia, National Cardiovascular Center Harapan Kita, Jakarta, Indonesia

**Keywords:** COVID-19, health personnel, health policy, occupational health, safety management, tricalcium phosphate, hydroxyapatite, fluoride, remineralization, microhardness

## Abstract

**Background and Aim:**

The coronavirus disease 2019 pandemic has brought deteriorating physical and mental burdens to health care workers (HCWs) in Indonesia, mainly attributed to the lack of protection and screening among HCWs, patients’ concealment of their travel and medical history, and perceived social stigma and discrimination. Hence, we deliver our perspectives and recommendations based on the current situation in Indonesia to enforce their safeties. We encourage stakeholders to implement a systematic approach by employing stringent prevention strategies, ensuring adequate personal protective equipment (PPE) provision and equitable PPE distribution, and routine HCWs screening to prevent nosocomial clusters, in addition to the provision of psychosocial support to HCWs by offering social aids and psychological sessions. Furthermore, social stigma and discrimination toward HCWs and patients should also be addressed and mitigated, thus preventing concealments of patients’ history and alleviating emotional burdens. We believe that providing continuous support to HCWs would lead to key benefits in ensuring a winning battle against the COVID-19 pandemic.

**Relevance for Patients:**

HCWs are pivotal players in winning the battle against the COVID- 19 pandemic. Ensuring their safety and well-being will enable them to deliver better healthcare services, thus resulting in mutual benefit for themselves, the patients, and the nation’s recovery.

## 1. Introduction

Until now, coronavirus disease 2019 (COVID-19) has afflicted global health burdens severely, infecting millions of people worldwide and resulting in hundred thousand of deaths [1]. During these moments, health care workers (HCWs) are among the most invaluable assets, risking their health caring for patients in needs. Despite their efforts, several concerns regarding their safety have arisen [2]. Hence, based on the current situation of HCWs in Indonesia, this article aims to deliver perspectives and recommendations to enforce the safety of HCWs during this pandemic.

## 2. COVID-19 in Indonesia: The Devastating Burdens to HCWs

Since early March 2020, COVID-19 has brought dreadful impacts on every aspect of human life. The cases of COVID-19 infection in Indonesia have exponentiated ever since, recording over 2 millions of cases and tens of thousands of deaths [3], indicating that Indonesia is still struggling to control the overwhelming burden of the COVID-19 pandemic.

The burden of the COVID-19 pandemic is even more pronounced in HCWs. Early evidence has indicated that a significant amount of COVID-19 infections was originated from nosocomial infections [4]. In Indonesia, nearly 1000 HCWs have fallen victim to the virus [5], rendering Indonesian as one of the countries with the highest COVID-19 mortality among HCWs [6]. This is further worsened by the fact that, despite rigorous efforts to accelerate COVID-19 vaccination among HCWs and general population [3], a recent COVID-19 case surge among vaccinated HCWs has been noted [5], thus further substantiating our premises.

One reason that may explain the high infection rate among HCWs in Indonesia is the fact that there have been several reported cases where concealment of patients’ travel and medical history had led to outbreak clusters among HCWs. In Yogyakarta, 53 HCWs were quarantined as a consequence of a patient being dishonest about his contact history, while another 57 HCWs had tested positive for the virus in Semarang due to travel history concealment [7]. This, coupled with the recent Ramadan exodus, raises concerns about potential clustering outbreaks affecting HCWs [8]. In addition, countless HCWs have been exposed to unnecessary risks due to minimal personal protective equipment (PPE), lack of COVID-19 screening among patients and HCWs, and physical and mental exhaustions due to long hour shifts [9-11]. All in all, these indicated that these issues should be promptly mitigated to prevent avoidable outbreak clusters.

In addition to physical burdens from risks of nosocomial infections, Indonesian HCWs are also afflicted by mental stress [12]. A recent report stated that over 80% HCWs in Indonesia has reported moderate physical and mental exhaustion due to the pandemic, half of whom required mental health support [5]. This may partly be caused by the prevalent human rights violations among Indonesian HCWs during the pandemic. Over 200 HCWs have reported that they had experienced stigma and discrimination in the communities during the COVID-19 pandemic from deprivation of essential services, social alienation, to physical assaults [4]. Some HCWs were even forced to leave their residences, albeit having tested negative [13]. Moreover, the communities’ distrust in HCWs and the public health system and the disbelief on COVID-19 further exacerbate the situation [12,14]. These events emphasize the urge to ensure the psychological well-beingness of HCWs in Indonesia by ensuring their human rights fulfillments both as a workforce and a human being.

## 3. Protecting HCWs in Indonesia: Perspectives and Recommendations

HCWs are among the population at highest risk of contracting the virus as they make regular contacts with patients. Since the beginning of the pandemic, they have worked for long shifts, thus increasing their vulnerabilities to physical and mental exhaustion [2]. Therefore, rigorous supports are required to ensure their well-beingness during these times. Herein, we highlighted three issues which aggravate the physical and psychological burdens to HCWs: (1) Minimum PPE and screening among HCWs, (2) patients’ dishonesty, and (3) stigma and discrimination.

### 3.1. Minimum PPE and screening among HCWs

Despite the urging from the government to prioritize PPE supplies for the HCWs, PPE shortage is relatively inevitable due to the overwhelming demands from both the health-care services and the public. Not to mention, the skyrocketing price of PPE complicates this condition even further as patients are reluctant to buy expensive standard PPE and difficulties in redistributing PPE to suburbs. Even after the implementation of rigorous guidance to enhance the efficiency of PPE usage, PPE shortages still persist leading to nosocomial infections [9,15]. It is essential to address this issue as adequate knowledge and compliant use of PPE was associated with lower COVID-19 risks [16]. This highlights the need to provide adequate supply of PPE to ensure that the HCWs at the frontline of health-care service are sufficiently protected.

These attempts essentially aim not only to fulfill supply chain orders by providing PPE but also to distribute PPE following critical equity framework. Recent evidences suggest that HCWs working on general wards may yield similar or even higher risks of contracting the virus when compared to HCWs attending COVID-19 specialized care facilities, mainly attributed to the inadequacy of protection [17]. Therefore, the equitable distribution of PPE is also important in addition to PPE provision itself. This paradigm will protect HCWs and prevent health system collapse in the short term, while also provide better understanding and empowerments to the functioning of HCWs and health systems in the society [17].

While it is true that adequate provision of proper PPE is essential to break the chain of COVID-19 transmission, prudent and thorough assessments are indispensable to ensure the implementation of ideal yet realistic approaches. Kampf *et al*. suggested that several alternatives may be implemented to replace the lack of alcohol-based hand rubs and gowns [18]. Furthermore, face masks may be worn prolonged or even reused following thermal disinfection or ultraviolet (UV) germicidal irradiation [18,19]. Derraik *et al*. stated that five disinfection cycles of N95 respirators may be achieved by applying heat of 70-75°C for 70 min or UVC light with a dose of 1500-2000 mJ/cm[2] per respirator surfaces. Surgical masks may also be similarly disinfected using heat treatment, as UV light may not be able to penetrate the deep mask folds [19]. While Kampf *et al*. suggested a lower temperature and a shorter time (i.e. 60°C for 30 min) [18], the opposite would be a safer option in inactivating the SARS-CoV-2 virus [20]. However, caution on prolonging the use of PPE should be placed as longer PPE use is associated with a higher incidence of dermatological side effects and a higher risk of non-adherent PPE behavior where HCWs tend to touch their PPE over time, thus increasing their susceptibility to the infection.

In addition, although disinfection of surgical gloves is unlikely to be the case, the use of medical gloves may be streamlined to adjust to the limited availability. This should first be achieved by minimizing the need of gloves by limiting their use to indicated procedures only [18,21]. Repeated usage of surgical gloves should be avoided whenever possible. However, if shortage persists, targeted disinfection of gloved hands for ongoing care on the same patient may be considered [18]. In any case, the principle of single and limited use of PPE is still recommended whenever possible, and the manufacturer should be contacted to ensure the efficacy and safety of such alternatives [18]. These approaches, along with robust supply chain and paradigm shift, may contribute to the mitigation of PPE issues, thus reinforcing the protection of HCWs. This is especially important considering that PPE inadequacy is one of the most reported cause of adverse psychological outcomes among HCWs [18].

In addition to inadequate PPE supplies, the lack of regular mass screening among HCWs also poses hazardous threats of nosocomial infections to other HCWs, and thus, depleting the number of available HCWs. As HCWs interact regularly with patients with comorbidities, it may also significantly increase morbidity and mortality rates to these vulnerable patients, considering the high proportion of asymptomatic COVID-19 cases [22]. Therefore, this addresses the importance of screening all HCWs on a regular basis to prevent nosocomial infections.

The mitigation of these issues requires a systematic and stringent approach to be integrated to the current infection prevention and control (IPC) practices. This should include: (1) obligations for HCWs with any respiratory symptoms to leave for at least 10-14 days until complete symptoms resolution [23,24], (2) separation of HCWs into teams of COVID-19 care and non-COVID-19 care [23,25], and (3) designation of sterile and COVID-19 contact areas [25]. Routine screening should also be enhanced by performing bi-daily temperature screening [25], bi-weekly serological tests [22], and confirmatory molecular tests if serological tests yield positive results [22], thus minimizing the risks of nosocomial infections from asymptomatic carriers. These strategies, if implemented accordingly, will minimize the risks of nosocomial clusters.

### 3.2. Concealment of patients’ travel and/or medical history

Patients’ cooperation during health-care provision is critical to prevent unnecessary infection clusters, and the history concealments pose imminent threats to HCWs and other patients. A recent study by O’Connor and Evans showed that patients contracting COVID-19 were more likely to conceal their social behavior [26], thus warranting investigations to infer potential causes leading to history concealments to better comprehend and countermeasure such an issue. During the COVID-19 pandemic, concealments of patients’ history are possibly caused by fear of potential social judgments and impactful social activity restrictions [27]. Social stigma on COVID-19 patients is attributed to the fact that COVID-19 is a relatively new and highly contagious disease and the nature of human’s fear to the unknowns, thus potentially creating baseless assumptions on COVID-19 patients. Consequently, COVID-19 patients may be labeled, stereotyped, or even alienated from the communities, which further aggravates the perceived fear and promote history concealments [28].

To tackle these issues, pragmatic approach should aim to protect both HCWs and patients. The prevention of history concealment should primarily address the source of exposure and reduce the social stigmata itself by clarifying misconceptions and implementing anonymous COVID-19 testing [27,28]. Furthermore, health systems should also be able to facilitate HCWs to confirm examination findings, possibly by enabling record linkage to travel or contact history [29]. The attempt of providing sufficient time to build rapport and educate patients about the risks and benefits of accurate contact tracing for suspected patients is absolutely feasible and necessary. Nevertheless, during pandemic, it is commonly acceptable to consider a symptomatic patient as a suspect despite insufficient contact history with confirmed cases to minimize transmission rate in the community and among HCWs. Finally, although law enforcements may contribute to prevent these concealments, such practices may be counterproductive as it relies largely on robust contact tracing system and may adversely affect patients’ psychological well-beingness [27].

### 3.3. Perceived social stigma and discrimination

Social stigma derived from the COVID-19 pandemic poses significant harm on patients and HCWs. Recent evidence indicates that Indonesian HCWs have suffered substantial emotional burdens both at work and home, experiencing symptoms of distress and depression due to the perceived stigma and fear of infecting their families [12]. These issues are important to address, considering that psychological burden can adversely impact HCWs’ resilience and work efficiency [27].

In this regard, alleviation of HCWs’ psychological burden should focus on addressing and removing the stigma. This may be achieved by enhancing solidarity and raising awareness among national and local communities through educational mass media campaigns to debunk misinformation and provide precise and accurate information related to COVID-19 and HCWs [30-32]. Notwithstanding the efforts required to carry out these measures, several reports have shown the potential benefits of these campaigns [31,33,34].

Provision of psychosocial support for HCWs can also alleviate their emotional burdens. The hospital management team should be aware of potential stressors exposed to HCWs and develop a plan to mitigate them. For example, setting proper working hours may improve their quality of life [35]. HCWs should be offered to participate in drop-in sessions to convey their concerns and be provided with peer supports as well as leisure activities to destress [35-38]. Peer supports should not only be limited to providing psychological aid but also to social support, which may aim to provide essentials and patronages to HCWs at work and in quarantine [36]. Furthermore, social support may also target HCWs’ families by establishing safe communication channels [35] and assisting childcare [39], as parenting stress and unmet childcare needs have been reportedly increased during this pandemic [39].

## 4. Conclusion

In the end, we believe that providing continuous support to HCWs would yield significant benefits during the battle against the COVID-19 pandemic. Specifically, we recommend that:

1. The principle of single and limited use of PPE to be preserved whenever possible. When such practice is not feasible, several alternatives to overcome the lack of PPE may be considered, provided that these alternatives are performed stringently and cautiously.
2. HCWs should be screened for COVID-19 on a regular basis to prevent nosocomial clusters. This practice should be equipped with robust PPE supply chain and systematic and stringent IPC to ensure the safety of HCWs during the COVID-19 pandemic.
3. Addressing and mitigating concealments of patients’ history and social stigma toward HCWs and COVID-19 patients should focus on enhancing solidarity and raising awareness among local and national communities. This may be achieved through educational mass media campaigns aiming to provide accurate information and debunk fake news on COVID-19.
4. HCWs should be provided with physical and psychosocial supports through the implementation of proper work hours, the mitigation of social stigma and discriminations, and the provision of adequate space for HCWs to alleviate their emotional burdens.

It is our greatest intention that these recommendations may help stakeholders to deliver appropriate policies to mitigate such issues.
